# Polarization Sensitive Optical Coherence Tomography for Bronchoscopic Airway Smooth Muscle Detection in Bronchial Thermoplasty-Treated Patients With Asthma

**DOI:** 10.1016/j.chest.2021.03.042

**Published:** 2021-03-27

**Authors:** Margherita Vaselli, Pieta C. Wijsman, Joy Willemse, Annika W.M. Goorsenberg, Fabio Feroldi, Julia N.S. d’Hooghe, Jouke T. Annema, Johannes F. de Boer, Peter I. Bonta

**Affiliations:** a Department of Physics and Astronomy, LaserLab Amsterdam, Vrije Universiteit de Boelelaan, Amsterdam, The Netherlands; b Department of Pulmonology, Amsterdam University Medical Center, University of Amsterdam, Amsterdam, The Netherlands

To the Editor:

Asthma is a chronic inflammatory disease characterized by airway remodeling (AR), including thickening of airway smooth muscle (ASM) as a key element of this alteration.^1^ Patients with severe asthma may benefit from bronchial thermoplasty (BT), an endoscopic treatment that targets structural AR and induces ASM reduction.^2^ Assessment of AR is critical to evaluate the impact of BT and might provide a tool for patient selection for BT. Polarization sensitive optical coherence tomography (PS-OCT) imaging has been proposed as a minimally invasive diagnostic method to assess ASM mass as an alternative to focal airway biopsies. Bronchoscopic standard optical coherence tomography (OCT) is an imaging modality that generates high-resolution cross-sectional images of the airways. PS-OCT imaging provides tissue-specific contrast by assessing tissue birefringence, which enables ASM detection and quantification.^3^ The aim of this pilot study is to evaluate in vivo bronchoscopic PS-OCT imaging as a safe and minimally invasive, volumetric imaging technique to visualize and quantify ASM. To our knowledge, this is the first time an ASM reduction after BT was detected by PS-OCT imaging and a correlation between ASM mass in biopsies and in vivo PS-OCT images was found. Moreover, 3-dimensional (3D) volumetric reconstructions of ASM before and after BT are performed.

This is a substudy of the TASMA (Unravelling Targets of Therapy in Bronchial Thermoplasty in Severe Asthma) trial,^4^ in which patients with severe asthma have been treated with BT, except for the middle lobe.^5^ In three patients, endobronchial PS-OCT imaging was performed in the right lower lobe (RLL) (four to seven pullbacks) and the untreated middle lobe (two pullbacks) 6 months after BT. One of these patients underwent bronchoscopy including PS-OCT imaging prior to BT treatment in the RLL (four pullbacks). PS-OCT imaging was found to be feasible and safe; no adverse events occurred. PS-OCT system specifications have been previously described.^6^ Volumetric PS-OCT images were acquired by inserting the OCT catheter (1.35 mm diameter) through the working channel of the bronchoscope into the selected airway segment until the alveolar compartment and helical scanning manual pullback was performed.

Analysis of 26 PS-OCT pullbacks (650-1,000 frames each) and statistical analysis of the data were done in MATLAB R2018a (MathWorks). To extract the birefringence properties of ASM, we adapted an algorithm developed by Villiger et al.^7^ Identification of the sample optic axis (OA), in which orientation is given by muscle fibers aligned in the same direction, provides a robust way of highlighting the presence of ASM.^5^ To retrieve depth-resolved OA orientation images, the presence of preceding birefringent layers^8^ and variation of the incident polarization state induced by the rotation of the catheter motor were addressed. To isolate birefringent structures in OA orientation images, the latter was thresholded with OA uniformity, as a measure of the uniformity of the OA orientation over a small region.^6^ ASM was automatically segmented from other birefringent layers by its consistent orientation throughout the pullback. For each airway segment, we measured the percentage of the ASM area over the total OCT cross section, extending from the outer sheath of the catheter to an imaging depth of 1.4 mm, and its average value along the pullback was calculated. Extremely distal frames imaged the alveolar compartment, where ASM was barely observed, and were not included in the calculations. Frames acquired proximally in the airway for which with the enlarging of the lumen ASM fall beyond the imaging depth of PS-OCT (airway diameter > 3 mm) were excluded.

Immediately after PS-OCT imaging, endobronchial biopsies were taken of predefined (sub)segmental airway carinas before and after BT treatment (two to four in the left lower lobe and RLL, and two in the distal and proximal middle lobe). Considering anatomic similarity between the lower lobes, biopsies from the nonimaged left lower lobe were included.^9^ In short, a total of 19 biopsies were paraffin-embedded, sectioned, and stained for ASM-specific desmin (clone-33; BioGenes); sections without epithelium/mucosal layer or with artefacts were excluded from the analysis.^10^ From each biopsy (except two), two sections with the highest surface area were included in the analysis and blindly measured, using automated digital image analysis software (ImageJ; NIH). ASM mass in the biopsies was measured as the percentage of positive stained desmin area compared with the total biopsy area.

**Figure 1:**
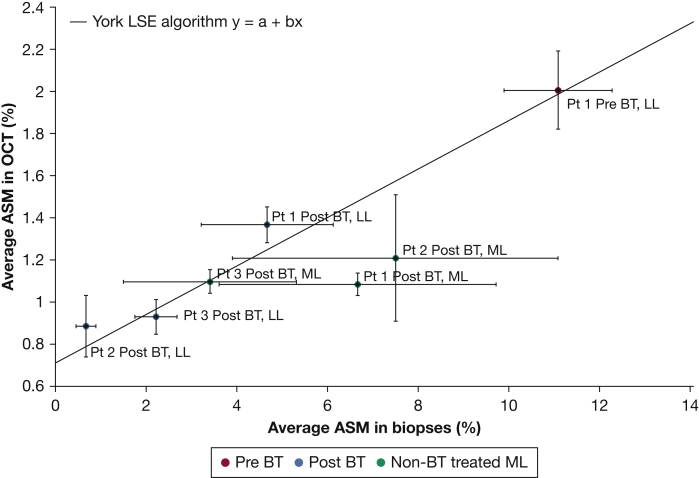
Histology-determined ASM content correlated with polarization sensitive optical coherence tomography (PS-OCT)-detected ASM content in LLs and non-BT treated ML airways of three imaged patients (Pts 1-3). Average ASM content in the biopsies was calculated as previously described and correlated with the average ASM content in the PS-OCT images. Error bar size is given by the SEM. A linear regression analysis based on York et al^11^ gave a statistically significant linear correlation between ASM content in PS-OCT imaging and in biopsies. P < .001 was found performing a χ^2^ test for the null hypothesis where the slope is zero. $York\;LSE\;algorithm:\;\chi^{2}\left(a,b\right)=\overset{N}{\underset{i=1}{\sum }}\frac{{\left(y_{i}-a-bx_{i}\right)}^{2}}{\sigma_{yi}^{2}+b\sigma_{xi}^{2}},\;\sigma_{\left(x,y\right)i}^{2}={\left(SEM\right)}_{\left(x,y\right)i}^{2}$ $\chi_{min}^{2}=2.68\Rightarrow \;a=0.71,\;b=0.115,\;\sigma_{a}=0.10,\;\sigma_{b}=0.023,\;P>.7$ $Null\;hypothesis:\;\chi^{2}\left(a=1.1,\;b=0\right)=39.90,\;P<.001\;$ A significant reduction in ASM mass after BT is visualized as the shift along the regression line for Pt 1 imaged before and after BT (data points Pt 1 Pre-BT LL and Pt 1 Post-BT LL). ASM = airway smooth muscle; BT = bronchial thermoplasty; LL = lower lobe; LSE = least square estimation; ML = middle lobe; OCT = optical coherence tomography; Pt = patient.

To investigate whether the PS-OCT-detected ASM content correlated with histology determined-ASM content, linear regression analysis including SEM as weights in both coordinates was implemented.^11^ Correlation of ASM content in multiple biopsies in the RLL and left lower lobe and PS-OCT pullbacks in the RLL was found. Performing a χ^2^ test (χ^2^ = 39.90, 5 *df*), an approximate value of *P* < .001 was found for the null hypothesis (slope is zero) (Fig 1). The larger SEM of the middle lobe biopsies was attributed to the significant difference of ASM content in distal and proximal biopsies.

**Figure 2:**
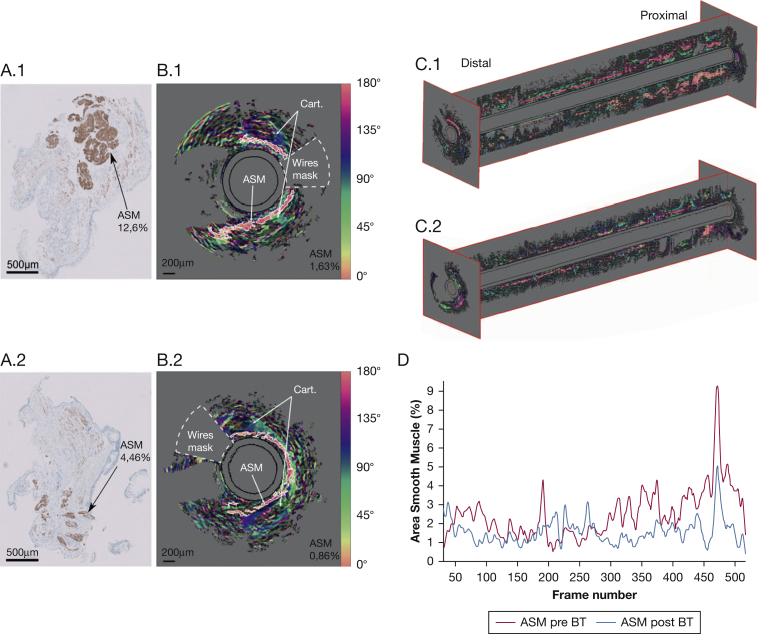
Airway biopsy histology and polarization sensitive optical coherence tomography (PS-OCT) images acquired in vivo in the right lower lobe airway segment of a patient with severe asthma before and after BT. Decrease in ASM content (%) after BT is observed in the desmin-stained biopsies (Fig 2A.1 before and Fig 2A.2 after BT). Relative optic axis (OA) orientation cross-sectional images of the same location before and after BT are shown in Figures 2B.1 and B.2, respectively. OA images have been thresholded with an OA uniformity value of 0.55 to isolate the ASM layer (pink) and two Cart. structures surrounded by birefringent connective tissue (green). The black circles represent the inner and outer edges of the plastic catheter sheath. The white dotted line delineates the tissue area not optically accessible because of the presence of the copper wires feeding current to the motor. OA cross-sectional images have been used to realize longitudinal reconstructions from part of the pullback (Fig 2C.1 before and Fig 2C.2 after BT, 550 frames). The amount of segmented ASM (%) in the airway has been plotted along the pullback in Figure 2D. ASM = airway smooth muscle; BT = bronchial thermoplasty; Cart. = cartilage.

To further substantiate that PS-OCT imaging is able to visualize and quantify ASM content, we investigated if PS-OCT imaging was able to detect a decrease in ASM content after BT in line with biopsy studies.^10^ A reduction of ASM was found performing an unpaired heteroscedastic *t* test (pre-BT: n = 4, post-BT: n = 5; *P* < .05) on PS-OCT measurements acquired in the patients imaged before and after BT. Reduction in ASM was visualized and quantified with PS-OCT images acquired before and after BT in the same airway location (Fig 2). The 3D volumetric reconstruction visualized a reduction of ASM especially in the proximal airway, which corresponds to the area directly treated with BT (Figs 2C, 2D). A decrease of ASM spikes was observed after BT in the proximal airway (Fig 2D). In general, a spiraling distribution of ASM along the airways was observed. The small number of patients imaged is a limitation of this study, but given the relatively high number of airways imaged before and after BT together with the histologic reference standard, we envision this work as a valuable proof of principle study. Future validation of this technique in a larger group of patients is needed. In conclusion, our data show bronchoscopic PS-OCT imaging is a promising minimally invasive 3D imaging technique to assess airway ASM content which might contribute to the assessment of AR in patients with severe asthma.
